# Experiences of care planning in England: interviews with patients with long term conditions

**DOI:** 10.1186/1471-2296-13-71

**Published:** 2012-07-25

**Authors:** Jenny Newbould, Jenni Burt, Peter Bower, Tom Blakeman, Anne Kennedy, Anne Rogers, Martin Roland

**Affiliations:** 1 Cambridge Centre for Health Services Research, University of Cambridge, Cambridge, UK; 2 Health Sciences Research Group, University of Manchester, Manchester, UK

**Keywords:** Aged, Chronic disease, Chronic illness, Patient care planning, Primary health care

## Abstract

**Background:**

The prevalence and impact of long term conditions continues to rise. Care planning for people with long term conditions has been a policy priority in England for chronic disease management. However, it is not clear how care planning is currently understood, translated and implemented in primary care. This study explores experience of care planning in patients with long term conditions in three areas in England.

**Methods:**

We conducted semi-structured interviews with 23 predominantly elderly patients with multiple long term conditions. The interviews were designed to explore variations in and emergent experiences of care planning. Qualitative analysis of interview transcripts involved reflexively coding and re-coding data into categories and themes.

**Results:**

No participants reported experiencing explicit care planning discussions or receiving written documentation setting out a negotiated care plan and they were unfamiliar with the term ‘care planning’. However, most described some components of care planning which occurred over a number of contacts with health care professionals which we term”reactive” care planning. Here, key elements of care planning including goal setting and action planning were rare. Additionally, poor continuity and coordination of care, lack of time in consultations, and patient concerns about what was legitimate to discuss with the doctor were described.

**Conclusions:**

Amongst this population, elements of care planning were present in their accounts, but a structured, comprehensive process and consequent written record (as outlined in English Department of Health policy) was not evident. Further research needs to explore the advantages and disadvantages of different approaches to care planning for different patient groups.

## Background

The prevalence and impact of long term conditions continues to rise. [1] Of particular concern is the escalation in multimorbidity, with an estimated two thirds of older people having two or more long term conditions. [2,3] People with these conditions frequently require complex care and support from a range of health and social care professionals over an extended time period. Long term conditions also demand much of patients if they and others around them are to meet the need for self-management effectively [4].

With increasing complexity and cost in caring for those with long term conditions, health and social care systems are challenged to find innovative, acceptable and appropriate approaches to meet their needs. This requires the reorientation of systems away from acute care to address the differing requirements of chronic longer term management. [5] Ham identified ten dimensions required in an effective health care system for long term conditions. [6] These include supporting patients to self-manage their conditions with help from informal carers; adopting population-management approaches such as the stratification of people with long term conditions according to risk and the provision of commensurate support; integrating care to facilitate primary care access to specialist advice and support; and coordinating care effectively.

The care planning approaches introduced by some health care systems in recent years are one way of achieving these wider goals. Care planning involves the negotiation and proactive arrangement of care between patients with a long term condition and their health and social care team. Key components of care planning include provision of information, shared decision making, and support for self-management. [7] Self-management support has been defined as “the facility that health care and social care services provide to enable individuals to take better care of themselves”. [8] The process of care planning may result in the output of a *care plan*, a written document summarising discussions and setting out agreed actions and goals.

As a new policy and form of practice, components of care planning present challenges for patients and professionals. Self-management support which is closely aligned to the principles of care planning can be important for patients, but may not be reflected in consultations. [9] Thus, both patients’ and professionals’ ways of articulating, prioritising and presenting conditions in consultations may not be conducive to care planning discussions, and topics such as emotional needs may be side-lined. [10,11] Further, GPs may experience tensions in their role in supporting patients to self-manage, and struggle with novel additions to the consultation [12].

The precise form and implementation of care planning varies between countries. In Australia, general practitioners who develop multidisciplinary care plans are remunerated, although uptake to date has been variable. [13,14] In a model used in Canada, patients’ requirements are evaluated across five areas (medication review, education and self-management, psychological and social assessment, social support, and prevention) and actions and goals recorded in a written care plan. [15] Within the UK, care planning approaches are set out within a Government personalisation policy agenda summed up in the slogan “no decisions about me without me.” [16] The NHS and Social Care Long Term Conditions model, based on the Kaiser Permanente Risk Pyramid, assigns patients to three tiered categories of risk: *case management* for patients with complex conditions; *disease management* for patients at medium risk; and *self-care support* for patients at low risk. [17] The importance of care planning for people with long term conditions at every tier is emphasised in the operating framework for the NHS in England 2010/11 [18].

**Figure 1:**
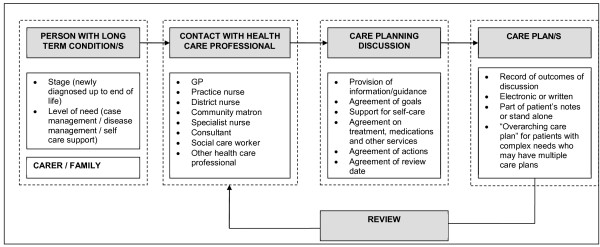
The care planning process as envisaged by English Department of Health policy.

Figure 1 summarises English Department of Health policy on care planning. Patients should participate in one or more explicit care planning discussions, covering the setting of personal goals, provision of information, support for self-management, agreements on treatment and medications, action planning, support services available and plans for future reviews. These discussions are intended to be recorded to form the patient’s written care plan. Those with particularly complex conditions may have an overarching care plan summarising agreements reached with multiple providers [19].

The introduction of the idea and practice of care planning is supported by disease-specific initiatives including Diabetes Year of Care [20] (a programme employing care planning alongside the commissioning of local services to improve self-management in diabetes) and the Co-creating Health Initiative [21] (a programme run by a non-governmental organisation aiming to improve self-management support and embed care planning within the clinical care of people with diabetes, COPD, persistent pain and depression). However, recent evidence from the General Practice Patient Survey shows that, whilst 84% of patients with long term conditions reported receiving care planning *discussions* within the last twelve months (defined as a discussion with a doctor or nurse about how best to deal with their long term condition), only 12% reported having a written care plan. [22] These results highlight the gap between policy and everyday clinical practice; it is not clear how care planning is currently implemented and embedded in primary care. This study draws on interviews with people with long term conditions to examine experiences of care planning discussions and care plans in England, and to explore barriers or facilitators to the implementation of this approach.

Broadly, we defined the process of care planning as involving:

· Anticipatory rather than reactive discussions about the patient’s care

· Defining roles and tasks among team members, including the patient

· Negotiating agreements that facilitate care within and across organisations

· Supporting patients to manage their own health and healthcare

· Promoting shared decision making

· Promoting care that is consistent with scientific evidence and the patient’s preferences

We had no preconceptions about what a care plan would look like or what time frame it would encompass, as we anticipated processes and documents would be variable based on local practice and patients’ needs.

## Methods

Interviews took place in two rural and one urban area in England as part of an evaluation of integrated care pilots. [23] Integrated care pilots aim to offer integrated care to meet local needs and priorities. The three integrated care pilots participating in the evaluation focused primarily on elderly, vulnerable patients at risk of hospital admission. The evaluation thus provided an opportunity to interview patients who, under English Department of Health policy, should have been offered care planning discussions by their health care professionals.

Sites applied to be integrated care pilots and were selected by the funder (the English Department of Health). Approaches to integrated care provision varied across the sites; Site A focused on co-ordinated care for elderly patients, Site B aimed to bring community and primary care working closer together and Site C had multiple work streams targeting specific populations, including the elderly. Interviews were conducted before integration activities began and therefore participants were receiving ‘usual care’ in each of the geographical areas.

In two of the three areas all patients who were part of the intervention were sent a questionnaire as part of the integrated care pilot evaluation (n = 200 patients in each area). In the third area (with a larger number of service users), a random sample (n = 500) were sent a questionnaire. The questionnaire included a tick box for respondents willing to be interviewed. 41% of questionnaire respondents at site A, 42% at site B, and 46% at site C indicated they would be happy to participate in an interview, from which our respondents were randomly sampled. Therefore, our sample comprised a range of predominantly elderly patients, with a variety of conditions, receiving standard care within each of the three participating areas.

Two researchers [JN and JB] conducted semi-structured interviews. The topic guide included specific questions on support for self-management, participation in decisions about care, coordination of care, and experiences of care planning and care plans. The guide was piloted and no amendments made.

We conducted interviews between April and June 2010 in participants’ homes, with the exception of one interview conducted at a local health centre. If present, informal carers were invited to participate. Interview length ranged from twenty-six minutes to one hour forty-six minutes, with a mean length of thirty-eight minutes. Interviews were audio recorded and transcribed verbatim. Permission to record was declined by one participant. Ethical approval was obtained from Cambridgeshire 3 research ethics committee (ref number 09/H0306/55).

Analysis followed the principles outlined by Lofland and Lofland. [24] These form a series of reflexive steps through which data are generated, coded, and re-coded, making particular use of memos to aid analytical thinking. Analysis was led by two authors [JN and JB]: all other authors contributed to the analysis by reading a selection of transcripts and offering reflections and suggestions on the developing coding framework. Codes were initially generated from the interview guide and literature. As coding progressed, we used memos to summarise and synthesise this work: memos were created for each new code assigned, as well as for potential themes or groupings of codes. Following initial coding across all data, we moved to a more focused period of iterative coding during which codes were rearranged, collapsed and sorted as we revisited the data and the literature. The computer program NVivo was used to assist with data management.

## Results

We conducted twenty three interviews; eight included both patient and carer (Table 1). Eleven patients were female and twelve male. Ages ranged from younger than fifty to over 85, with most aged over 75. Participants reported between two and nine health problems. Conditions were varied and often severe: they included chronic back pain, cancer, diabetes, arthritis, COPD and heart problems.

**Table 1:** Respondent socio-demographic details

**Respondent number**	**Gender**	**Age**	**Relationship status**	**Area**	**Conditions as described by patients**
1-1	Female	65-74	Widow	Rural	Cancer, heart problems
1-2	Female	75-84	Married	Rural	Osteoporosis, osteoarthritis, thyroid problems, eye condition
1-3	Male	65-74	Married	Rural	Stroke, osteoarthritis, back problems,
1-4	Male	Under 65	Married	Rural	Multiple sclerosis, diabetes
1-6	Female	75-84	Widow	Rural	Osteoarthritis, heart problems, eye condition
1-7(joint interview)	Male(&female)	75-84	Married	Rural	Cancer, asthma, COPD, osteoporosis
1-8	Female	75-84	Widow	Rural	Heart condition, back problems
1-10(joint interview)	Male(&female)	75-84	Married	Rural	Cancer, heart condition, eye condition, diabetes
1-11	Male	75-84	Single	Rural	Diabetes, memory loss
2-1(joint interview)	Male(&female)	75-84	Married	Town	Heart condition, foot condition, knee problems
2-2	Male	75-84	Widower	Town	Heart condition, prostate problems
2-3(joint interview)	Male(&female)	75-84	Married	Town	COPD, cancer
2-4	Female	65-74	Widow	Town	Heart condition, cancer, asthma, osteoporosis
2-5	Female	Over 85	Widow	Town	Heart condition, osteoporosis, osteoarthritis
2-6(joint interview)	Male	75-84	Married	Town	Heart condition, neuralgia
2-7(joint interview)	Male(&female)	75-84	Married	Town	Heart condition, prostate problems
2-8	Female(&male)	75-84	Married	Rural	Stroke, eye condition, thyroid problem, heart condition
					Male: Eye condition, osteoarthritis
2-9(joint interview)	Male(&female)	75-84	Married	Rural	Male: Heart condition, osteoporosis
					Female: Cancer, heart condition
2-10	FemaleOver	85	Single	Town	Back problems, heart condition
3-1	Male	Under 65	Single	Rural	Cancer, mental health condition
3-2(joint interview)	Female (& male)	75-84	Married	Rural	Female: Liver condition, diabetes
3-3	Female	75-84	Divorced	Rural	Asthma, COPD, heart condition, kidney condition
3-4	Female	Over 85	Married	Town	Diabetes, dementia, mental health condition

No participants reported experiencing distinct and explicit care planning discussions, as defined earlier, or receiving written documentation setting out a negotiated care plan. They were unaware of the term care planning and what it might involve. Some components of care planning were, however, identifiable in participants’ reports of care. We outline the nature of these components, along with barriers to their effective implementation.

### The reactive nature of care planning

Most participants had some form of discussion with their health care professionals which included components of care planning and was aimed at assisting them in managing their condition better. These discussions were not overtly designated care planning meetings; instead they occurred throughout participants’ routine and emergency contacts with health care professionals, primarily GPs but also hospital doctors and nurses. Matters of chronic illness management were therefore dealt with on a predominantly reactive basis with a variety of providers over a period of time (see Table 2 for an illustrative case summary).

**Table 2:** Illustrative case study: John and Sheila

**ID**	**Respondent story**
2-3	John (not his real name) is on oxygen 15 hours a day, and struggles to breathe; he attributes this to asthma. He has previously had surgery and radiotherapy for lung cancer, and also has bowel problems for which he takes medication. He attends hospital to see “the oxygen nurse” and “the cancer doctor”. He has had multiple admissions to hospital in recent years for chest infections. A nurse visits at home to give injections when required, and to see if they have everything they need, but they are not sure of her specific role. His wife Sheila (not her real name) had a hip replacement operation two year ago, and has osteoarthritis. She helps care for John who, for example, requires some help getting dressed. They had carers visit immediately after his cancer surgery, but this was a short-term arrangement, and Sheila struggles to keep up with the shopping and household jobs. He and his wife visit the GP *“just if we need*”, or as John said, *“there has to be something wrong.”* They prefer one GP in particular but, visiting as they do usually on an acute basis, they do not often manage to get an appointment with her. The only written information John has received to date and still refers to he calls his “rules and regulations”. This is a set of exercises and management techniques given to him by a respiratory nurse to help with his breathing – as his wife says “*And if he does them he’s a lot better. But then he thinks he’s a lot better so he doesn’t do them.”* They have never sat down with one health care professional to review treatment, care and medication or discuss plans for the future. Instead, they have gleaned what information they wish to have from a wide variety of sources, including hospital doctors and nurses, physiotherapists, various community nurses and GPs. If they have questions about an issue or concern, they might consider going to see the GP, but they rarely do so as it is difficult to get an appointment and they feel there is not much time to discuss issues. Recently a hospital doctor suggested keeping prophylactic antibiotics and steroids in the house for when his chest gets bad again, but they do not know what is happening about that. They are still confused about some aspects of John’s current medication, which has changed recently. Asked if they wished to discuss anything else concerning their healthcare, they said:John: No I don’t think so, no. No, we’re alright aren’t we?*Sheila: Yes, we’re alright as long as I can keep going.*John: Aye and I can keep going.*Sheila: And you can keep going. You can keep breathing yes.*

"Oh he (GP) did, we sat down and discussed it all, showed me, he drew little pictures and things and of course he knew my nursing background so he was able to explain it all and I was able to understand."

Participants rarely reported discussions about care covering the full range of recommended care planning components, including the provision of information, support for self-management and the coordination of care (see Figure 1);

"I: Do you ever feel that you’ve had the opportunity to discuss, say, with your GP, sort of an overall view of your healthcare and your health and how you’re managing that?"

"R: No, I don’t think so. Not a lot of what the future might hold, he just says, well, this could improve a bit or it’s definitely going to get worse."

### Communication of information

Experiences of the communication of information by professionals varied. Many participants felt professionals provided, overall, sufficient information about their condition, although understanding was typically built up over a number of years [See Table 2].

However, some described a frustrating lack of information, with two stating health care professionals appeared unsure about their condition or appropriate treatment.

Perceptions of the effective flow of information between professionals and patients were linked to what was considered continuity of care. Many participants wanted to see a GP who knew them, as a lack of continuity meant a perceived lack of understanding;

"… All he (locum GP) will have is the information on his screen which okay I mean technology as we know is marvellous it can, he can learn a lot off that screen but if he is only there for a week or a fortnight there is never going to know looking at that screen what 20 years. I mean if you were a doctor and I was your patient, if you saw me for 20 years you would know every single thing and you would know when I was trying to con you…"

One participant who had experienced years of chronic back pain felt her condition was trivialised by GPs unfamiliar with her case;

"… It’s frustrating actually, you know, because sometimes you get on and you think, oh God, here’s another one [GP], you know, now I’ve got to start from scratch. I mean, they come in to me and say, “so you’ve a bad back love, haven’t you?” And you want to scream. You literally want to scream at them."

Perceived limitations on consultation time also curtailed the opportunities for information exchange;

"It is time pressured. Because really, you only get about 5–10 minutes with your GP, and you’ve got to be right on the thing. Like I’m talking to you, I haven’t had the time to discuss with her things like that, I just go in, tell her what my problems are and how I am. I realise that she’s busy, she’s a lot of other patients to see. So you can’t take that much time."

### Support for self-management

A number of participants, usually those who felt they had sufficient information and experience, indicated that they were confident managing their health problems, although often with assistance from paid or unpaid carers. Many were practiced in daily routines including monitoring blood glucose and managing complex medication. Professional assistance with aspects of self-management was evident, for example the advance provision of antibiotic supplies or simple encouragement;

"But if I’m getting windy about it, and sort of a bit cowardly, I will ring up the district nurse, and she’ll say, ‘just carry on Edward’, and she gives you those words of reassurance."

For many, self-management was more concerned with achieving important everyday activities than with specific medical tasks. These included learning to walk without a stick so they could carry a cup of tea or getting out for fresh air;

"It’s a very good Zimmer but you trundle along with it and then you put the brakes on and you sit down, which is marvellous because if I sit down I’m fine. Then I’m up again and do another bit and so I try, every day, and get out a little bit, down to the bottom and back."

A number of participants were trying to cope with psychological elements of their condition, such as the impact of physical limitations and changes, and the perceived need to remain positive for the sake of others. However, these goals tended to be supported by informal carers alone; participants rarely discussed psychological concerns with their health care professionals.

Only three participants mentioned that ‘goal setting’ and ‘action planning’ were discussed at all in relation to their care. One respondent did not remember the process of goal setting other than the term. The two other participants felt the terms were not applicable given their conditions;

"I: And who tends to talk to you about goal setting?"

"R: Usually the Nurse and the Doctor says (about it) there is not much point in that when you can’t walk is there. Which there isn’t, is there?"

"I haven’t really got an action plan....I don’t think you can have an action plan when you’re in my condition of life [paralysed from the neck down]. My action plans is just to try and live life to the full."

During interviews participants often raised concerns about the future of their condition or care. Anxieties included the implications of a hospital stay for further care, a lack of knowledge about available home care support and concern at having to leave their current home if their condition deteriorated;

"But I don’t like the concept of, such as myself, having to go into a home as an alternative. I don’t like that at all. I would hate to leave this little flat and I would do anything to organise help to be able to stay and I think a lot of people would be far happier if that was so."

Participants believed such topics were not issues of concern for health care professionals, and thus rarely mentioned these aspects in consultations.

### Coordination of care

Many participants had multiple conditions requiring complex treatment and coordination between themselves, their carers, and a number of professionals across primary and secondary care. Patients noted phone conversations, face to face meetings and letters between health care professionals as forms of communication they were aware of;

"It seems to be working alright. This doctor I see at (hospital) sends letters to my doctor (GP), and my doctor is in touch with them about various things. I’ve just had, a pharmacist across the road, and he’s sent a form to my doctor about this thing. It’s just like a form, informing about my medication that’s going on…"

However, five participants felt that care was not co-ordinated well across different teams and that this was something that was a matter for the health care delivery team to manage over which they had little or no control;

"When you go to the orthopaedic department the consultant that does the arms won’t touch legs. A leg man won’t touch the spines, you know the spine man won’t touch the hip and you have to be referred back to your doctor who then refers you back to another doctor, another consultant and you try to explain to him mixture between the two, he doesn’t want to know because he only does legs, he doesn’t do back… But as you can see is a very difficult situation for the white coats as I call them to understand when they don’t want to talk amongst themselves even in the same department. It’s very difficult."

Patient-related barriers to comprehensive treatment discussions included an unwillingness to engage with their condition – as one respondent (2–4) commented, “*I bury my head in the sand as far as my heart’s concerned.”*

For a small number, this reluctance to discuss their care and conditions had arisen from the perception that nothing more could be done for them;

"“Mr Jones,” he said, “as I’ve written down, there’s no hope”.... You know we said to him, “You know what is the prognosis?” He said, “You go back home, you go back to bed and you stay there until the end”."

Such conversations were identified as ending any future discussions about care: if there was nothing that could be done, there was no point in planning ahead. As a result, some patients had retreated from seeing professionals unless absolutely necessary.

### Written documentation

Only one participant recalled a document which he identified as a care plan, although he had not looked at it;

"Oh yes, the nurses always write you a care pack…Oh yeah, there is a care plan yeah. No, that’s, they do fill in a, because that’s just - I don’t see it as such because, my wife will know where it is and when the nurses come in, the doctor will read that sometimes… And he will, my doctor will read what, like if I’m being dealt with for a pressure sore."

However, from the description above, this is more likely to be a nursing/shared record rather than documentation of the outcome of care planning discussions. Another respondent described completing forms with a nurse about her wishes for end of life care. She did not believe this to be a care plan, but it may have contained elements of advanced care planning.

## Discussion

### Summary of main findings

**Figure 2:**
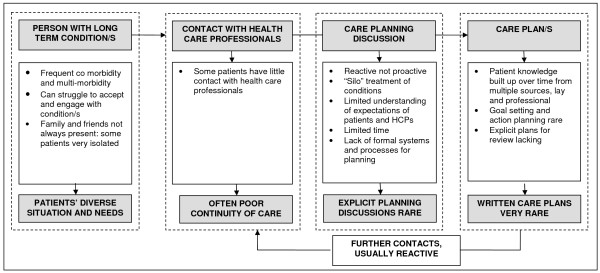
“Reactive” care planning as experienced by study participants.

In this predominantly frail elderly population, care planning was not a discrete event with an explicit discussion leading to a written care plan, as envisaged by policy (Figure 1). Instead, respondents experienced elements of care planning through different healthcare contacts, in what we term “reactive” care planning (Figure 2). In this reactive rather than proactive approach, care planning was often inconsistent and incomplete, and explicit goal setting and action planning were rare. Poor continuity and coordination of care, lack of time in consultations, and patient attitudes towards legitimate discussion topics with health care professionals, may be key barriers to care planning.

### Strength and limitations of the study

We conducted a small number of patient and carer interviews. We did not interview their health care professionals to provide a useful additional perspective. Interviews took place within a larger study of integrated care pilots, restricting participants to those who lived within an integrated care pilot area. Participants were drawn from predominantly rural areas, with a mixture of village and town residence, and their experiences of health care may differ from those living in urban areas. Ethnicity was also predominantly white. However, the range of conditions and multimorbidity experienced by participants is broadly representative of the wider community of patients with long-term conditions.

Health literacy, conceptualised as a personal asset, is orientated to developing skills and capacities which enable individuals to exert more mastery and control over their health and the factors that influence health and illness. [25] This may be an important component of patient’s ability to participate in health care, including the care planning process. [26] However, we did not collect information on participant’s educational attainment as an indicator of health literacy. [27] Medical records would provide further detail on participants’ conditions and communication and coordination between health care professionals. However, we drew participants from GP practices across three geographical areas, representing a sample of older patients with multiple long-term conditions experiencing standard care.

### Comparison with existing literature

Patients identified GPs as the person with whom discussions about care primarily occurred. GPs and patients with serious conditions may particularly value a personal relationship, [28] and we identified poor continuity of GP care as a significant barrier to effective information exchange. Where the development of understanding about conditions and goals occurs over a period of time, rather than being confined to discrete “care plan” consultations, continuity and being able to see the provider of choice when required are likely to be more important factors in the successful implementation of care planning approaches [29].

English Department of Health policy identifies support for self-management, goal setting and action planning as key care planning components. We found little evidence of shared goal setting and action planning. Some respondents were competent self-managers, but many expressed anxiety about social and practical as well as medical aspects of their condition. Their focus on self-management as everyday activities rather than medical tasks may lead patients to question the relevance of goal setting and action planning, as these may be perceived as concerned with medical matters which remain the domain of health care professionals. [30] However, it is of concern that participants felt social and emotional matters were inappropriate to raise with health care professionals, as care planning aims to facilitate self-management across all dimensions of a person’s life. This may be compounded by health care professionals’ reluctance to initiate self-management discussions. [10] It is possible that cultural expectations regarding care planning have yet to be established amongst both patients and health care professionals. Ideas around the role of the patient and the provider within long term conditions, and the purpose of the consultation, may have yet to re-orient to enable explicit care planning processes to be given sufficient attention.

The documentation of discussions in a written care plan is strongly supported by current policy. As none of our participants had participated in explicit care planning discussions, none had a written document. Written documentation may be useful if care planning is to be sustained over a period of time with a number of professionals, or when continuity is more difficult to achieve. It may also function to maximise the impact of care planning, enhancing behaviour change by providing a record which can be used to self-monitor progress, and to specify agreements between patients and professionals. [31] However, current evidence on the effectiveness of written action plans for conditions including asthma and COPD is mixed [32,33].

The rarity of proactive care planning in this patient group may reflect, in part, a corresponding absence of systems to support care planning and the production of care plans. For self-management support a whole systems perspective that engages patient, practitioner, and service organisations has been advocated. [34] Whilst English policy has emphasised the importance of the care planning approach, it has not provided specific changes to systems to implement this. Previous research in diabetes care has found that integrated electronic health records can help to promote coordination of care, although patients with more complex needs may require innovative structured communication strategies to support care planning. [35] Programmes such as the diabetes-specific Year of Care emphasise the importance of system re-design to facilitate care planning. [20] It is possible that care planning and the production of care plans is perceived (by patients and/or health care professionals) to be incompatible with or disruptive to usual practice; if this is the case, carefully designed systems to support the implementation of this approach will be required.

### Implications for future research

We have identified a reactive model of care planning that diverges from suggested approaches outlined within current policy. Further research needs to explore the advantages and disadvantages of different approaches for different patient groups. All participants in this study had more than one condition. To date much care planning work in the UK has been disease specific, and there is little evidence on the implementation or function of care plans for patients with more complex needs. Certainly, they could provide an important platform to coordinate care for patients with multimorbidity, making responsibilities and lines of communication explicit. [36] Our data suggests patients may welcome discussions about and support for wider concerns. To achieve this, it may be necessary for professionals to engage these patients more directly in care planning. A focus on professionals’ attitudes to and use of the care planning approach is also required in order to develop understanding of the likely supply side barriers to effective adoption and embedding of this approach in daily practice.

## Competing interests

The authors declare that they have no competing interests.

## Authors’ contributions

JN and JB carried out the interviews, analysed the data and drafted the manuscript. PB and MR conceived the study and participated in its design and coordination and helped to draft the manuscript. AR participated in the design of the study, contributed to the analysis of the data and helped to draft the manuscript. TB and AK and participated in the design of the study and contributed to the analysis of the data. All authors read and approved the final manuscript.

## Pre-publication history

The pre-publication history for this paper can be accessed here:

http://www.biomedcentral.com/1471-2296/13/71/prepub

## References

[B1] WHO Preventing Chronic Diseases A vital investment 2005 WHO

[B2] Wolff JL Starfield B Anderson G Prevalence, expenditures, and complications of multiple chronic conditions in the elderly Arch Intern Med 2002 162 20 2269 2276 10.1001/archinte.162.20.2269 12418941

[B3] van den Akker M Buntinx F Metsemakers JF Roos S Knottnerus JA Multimorbidity in general practice: prevalence, incidence, and determinants of co-occurring chronic and recurrent diseases J Clin Epidemiol 1998 51 5 367 375 10.1016/S0895-4356(97)00306-5 9619963

[B4] Sobel DS Rethinking medicine: improving health outcomes with cost-effective psychosocial interventions Psychosom Med 1995 57 3 234 244 7652124 10.1097/00006842-199505000-00005

[B5] Nolte E McKee M Nolte E, McKee M Caring for people with chronic conditions: an introduction Caring for people with chronic conditions: A health system perspective edn 2008 Maidenhead: Open University Press

[B6] Ham C The ten characteristics of the high-performing chronic care system Health Econ Policy Law 2010 5 Pt 1 71 90 19732475 10.1017/S1744133109990120

[B7] Personalised Care Planning[http:\\www.dh.gov.uk/prod_consum_dh/groups/dh_digitalassets/documents/digitalasset/dh_124048.pdf]

[B8] Osborne RH Jordan JE Rogers A A critical look at the role of self-management for people with arthritis and other chronic diseases Nat Clin Pract Rheum 2008 4 5 224 225 10.1038/ncprheum0765 18301410

[B9] Morris RL Sanders C Kennedy AP Rogers A Shifting priorities in multimorbidity: a longitudinal qualitative study of patient’s prioritization of multiple conditions Chronic Illn 2011 7 2 147 161 10.1177/1742395310393365 21343220

[B10] Blakeman T Bower P Reeves D Chew-Graham C Bringing self-management into clinical view: a qualitative study of long-term condition management in primary care consultations Chronic Illn 2010 6 2 136 150 10.1177/1742395309358333 20444765

[B11] Wilson PM Kendall S Brooks F The Expert Patients Programme: a paradox of patient empowerment and medical dominance Health Soc Care Community 2007 15 5 426 438 10.1111/j.1365-2524.2007.00701.x 17685988

[B12] Blakeman T Macdonald W Bower P Gately C Chew-Graham C A qualitative study of GPs' attitudes to self-management of chronic disease Br J Gen Pract 2006 56 527 407 414 16762121 PMC1839014

[B13] Zwar NA Hermiz O Comino EJ Shortus T Burns J Harris M Do multidisciplinary care plans result in better care for patients with type 2 diabetes? Aust Fam Physician 2007 36 1–2 85 89 17252093

[B14] Harris MF Jayasinghe UW Taggart JR Christl B Proudfoot JG Crookes PA Beilby JJ Davies GP Multidisciplinary Team Care Arrangements in the management of patients with chronic disease in Australian general practice Med J Aust 2011 194 5 236 239 21381995 10.5694/j.1326-5377.2011.tb02952.x

[B15] Russell G Thille P Hogg W Lemelin J Beyond fighting fires and chasing tails? Chronic illness care plans in Ontario, Canada Ann Fam Med 2008 6 2 146 153 10.1370/afm.793 18332407 PMC2267432

[B16] Department of Health Equity and excellence: Liberating the NHS 2010 London: Department of Health

[B17] Department of Health Supporting People with Long Term Conditions: An NHS and Social Care Model to support local innovation and integration 2005 London: Department of Health

[B18] Department of Health The Operating Framework for the NHS in England 2010/11 2009 London: Department of Health

[B19] Department of Health Supporting People with Long Term Conditions: Commissioning Personalised Care Planning: a guide for commissioners 2009 London: Department of Health

[B20] NHS Diabetes Year of Care[http://www.diabetes.nhs.uk/year_of_care]. Accessed 22 July 2012

[B21] The Health Foundation Co-creating Health[http://www.health.org.uk/areas-of-work/improvement-programmes/co-creating-health/]. Accessed 22 July 2012

[B22] Burt J Roland M Paddison C Reeves D Campbell J Abel G Bower P Prevalence and benefits of care plans and care planning for people with long-term conditions in England J Health Serv Res Policy 2012 17 Suppl 1 64 71 22315479 10.1258/jhsrp.2011.010172

[B23] Europe RAND Ernst and Young LLP: National Evaluation of the Department of Health’s Integrated Care Pilots 2012 Cambridge

[B24] Lofland J Lofland LH: Analyzing Social Settings 1995 A guide to qualitative observation and analysis. Belmont: Wadsworth

[B25] Nutbeam D The evolving concept of health literacy Soc Sci Med 2008 67 12 2072 10.1016/j.socscimed.2008.09.050 18952344

[B26] Protheroe J Nutbeam D Rowlands G Health literacy: a necessity for increasing participation in health care Br J Gen Pract 2009 59 567 721 723 10.3399/bjgp09X472584 19843420 PMC2751916

[B27] Ishikawa H Yano E Patient health literacy and participation in the health-care process Health Expect 2008 11 2 113 122 10.1111/j.1369-7625.2008.00497.x 18494956 PMC5060442

[B28] Kearley KE Freeman GK Heath A An exploration of the value of the personal doctor-patient relationship in general practice Br J Gen Pract 2001 51 470 712 718 11593831 PMC1314098

[B29] Shortus THM Groves MFJ Optimising Health Outcomes in Chronic Disease Communication Skills in Medicine: Promoting Patient-Centred Care 2010 Melbourne, Australia: IP Communications 48 61

[B30] Lorig K Holman H Self-management education: History, definition, outcomes, and mechanisms Ann Behav Med 2003 26 1 1 7 10.1207/S15324796ABM2601_01 12867348

[B31] Abraham C Michie S A taxonomy of behavior change techniques used in interventions Health Psychol 2008 27 3 379 387 18624603 10.1037/0278-6133.27.3.379

[B32] Toelle B Ram Felix SF Written individualised management plans for asthma in children and adults Cochrane Database of Systematic Reviews 2004 John Wiley & Sons, Ltd 10.1002/14651858.CD002171.pub2 15106169

[B33] Turnock Allison C Walters EH Walters Julia AE Wood-Baker R Action plans for chronic obstructive pulmonary disease Cochrane Database of Systematic Reviews 2005 Chichester, UK: John Wiley & Sons, Ltd 10.1002/14651858.CD005074.pub2 16235392

[B34] Kennedy A Rogers A Bower P Support for self care for patients with chronic disease Br Med J 2007 335 968 970 10.1136/bmj.39372.540903.94 17991978 PMC2071971

[B35] MacPhail LH Neuwirth EB Bellows J Coordination of Diabetes Care in Four Delivery Models Using an Electronic Health Record Medical Care 2009 47 9 993 999 10.1097/MLR.0b013e31819e1ffe 19648836

[B36] Bower P Macdonald W Harkness E Gask L Kendrick T Valderas JM Dickens C Blakeman T Sibbald B Multimorbidity, service organization and clinical decision making in primary care: a qualitative study Fam Pract 2011 28 5 579 587 10.1093/fampra/cmr018 21613378

